# Supplementary figures and images for: BRAFV600E induces reversible mitotic arrest in human melanocytes via microRNA-mediated suppression of AURKB
Source: eLife. 2021 Dec 14;10:e70385. doi: 10.7554/eLife.70385 (PMC8610417; doi:10.7554/eLife.70385)

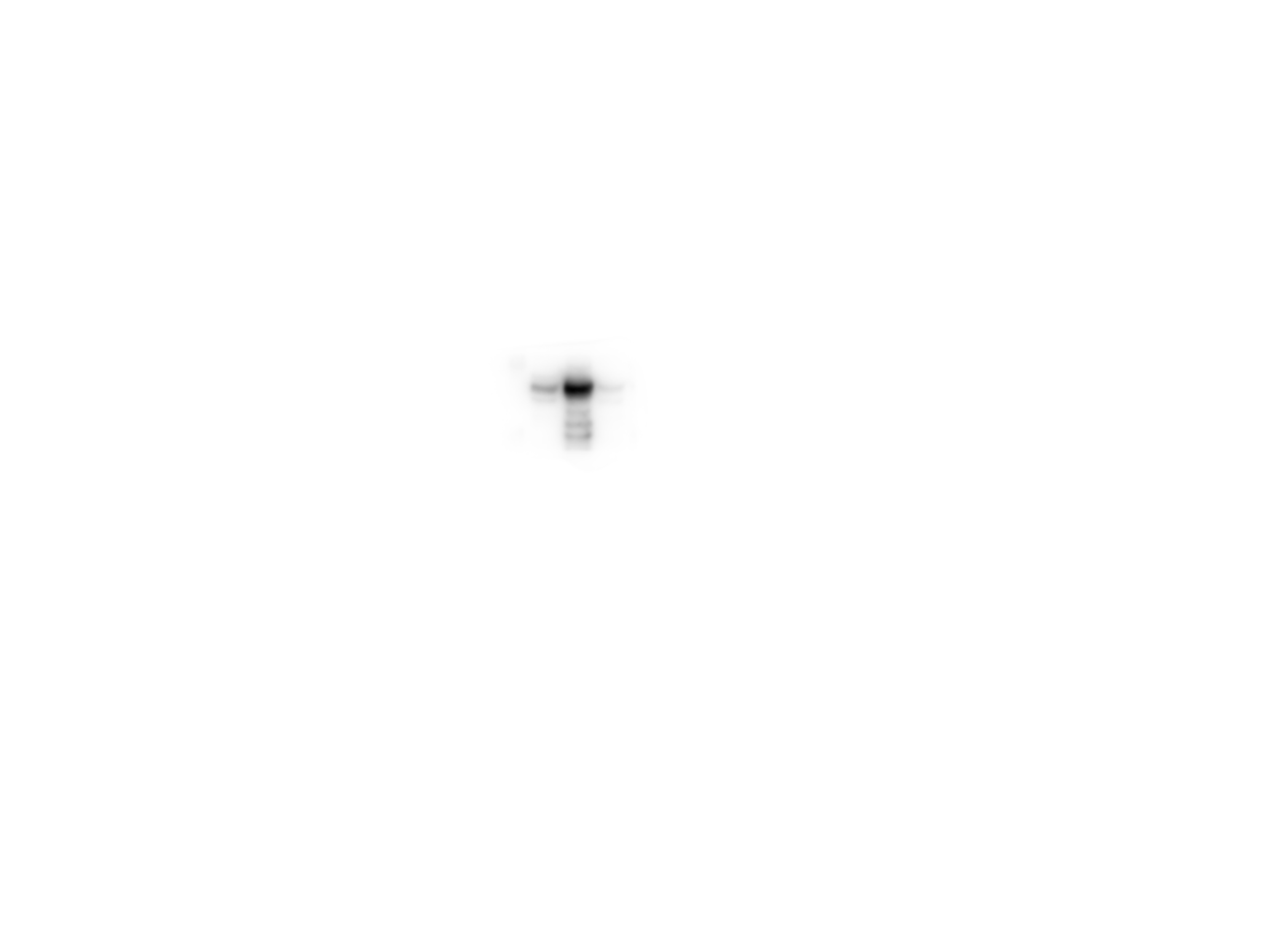

Supplement: Figure 2—source data 1. [file elife-70385-fig2-data1.zip › aurkb_wb/supp_fig_S2_source_data_aurkb.tif]

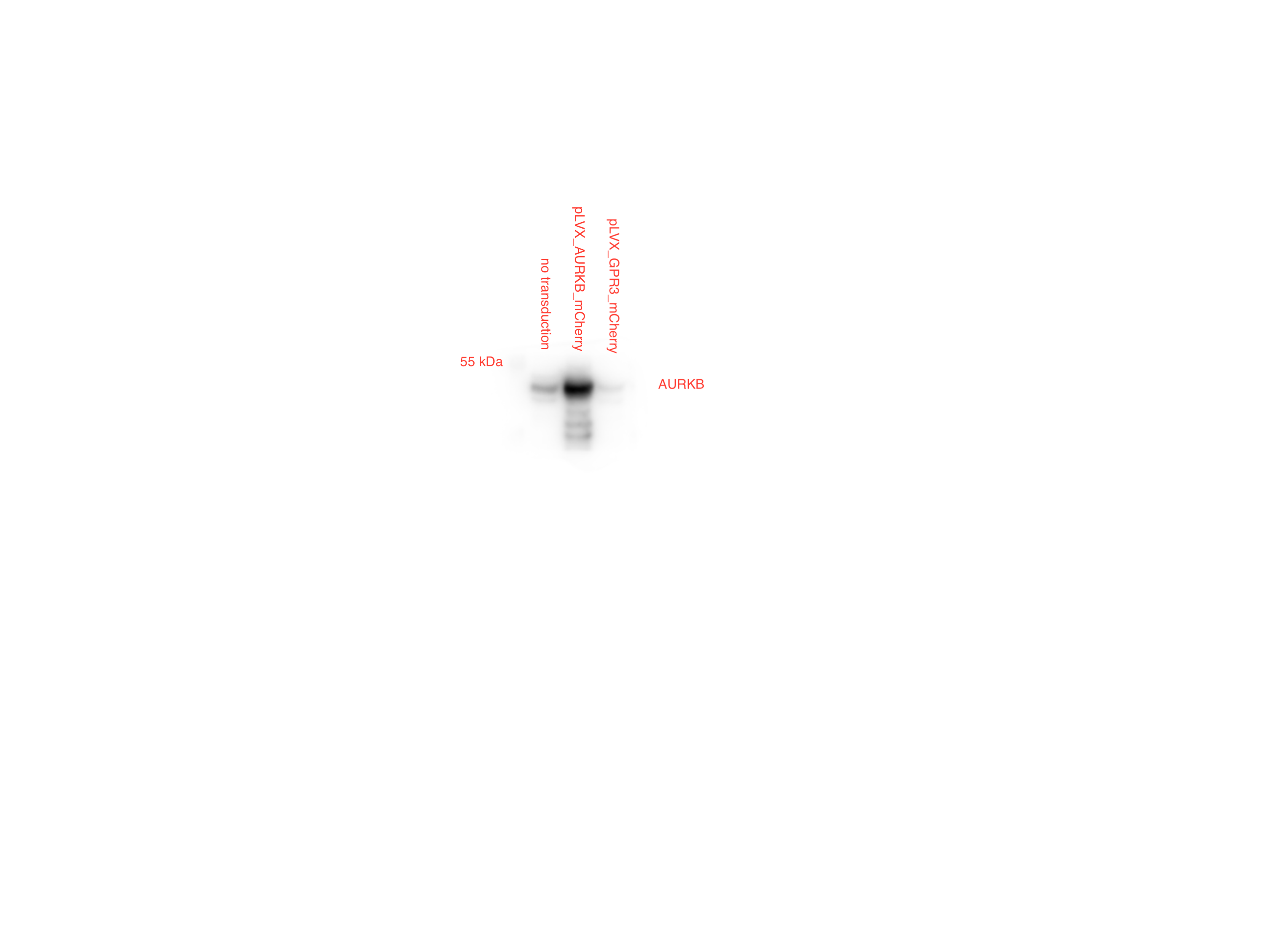

Supplement: Figure 2—source data 1. [file elife-70385-fig2-data1.zip › aurkb_wb/supp_fig_S2_source_data_aurkb_annotated.tiff]

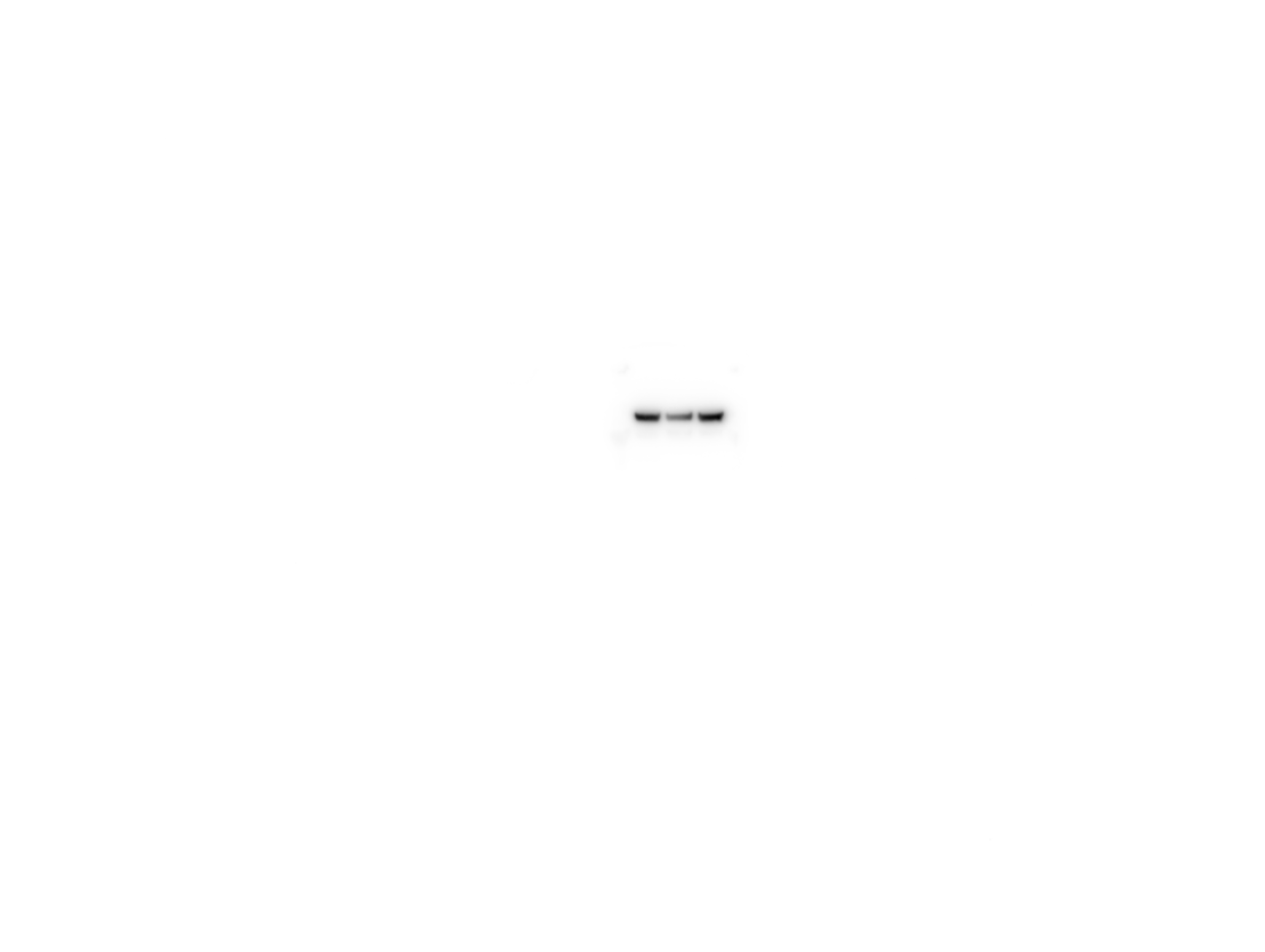

Supplement: Figure 2—source data 1. [file elife-70385-fig2-data1.zip › aurkb_wb/supp_fig_S2_source_data_hsp90.tif]

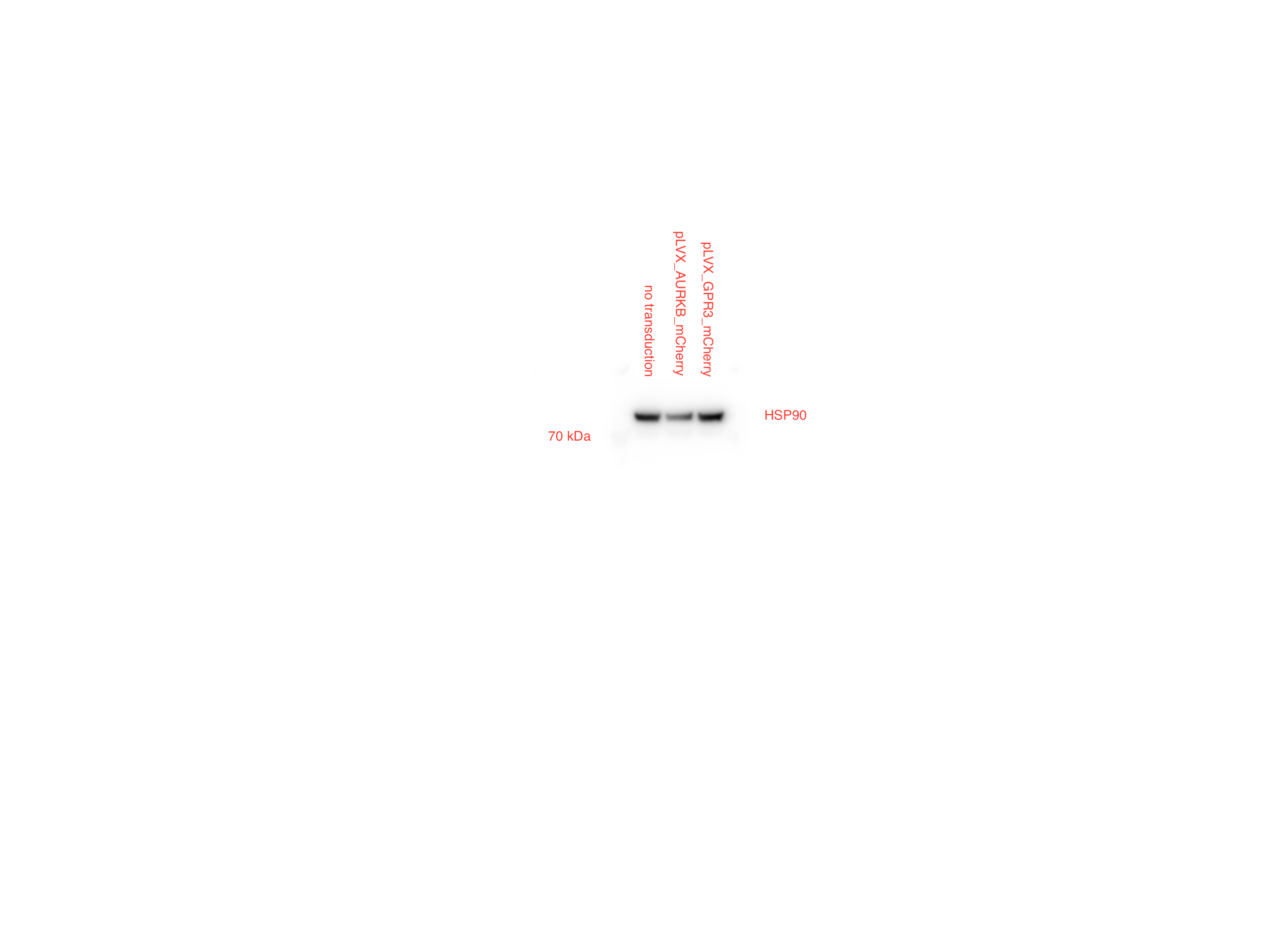

Supplement: Figure 2—source data 1. [file elife-70385-fig2-data1.zip › aurkb_wb/supp_fig_S2_source_data_hsp9_annotated.tiff]

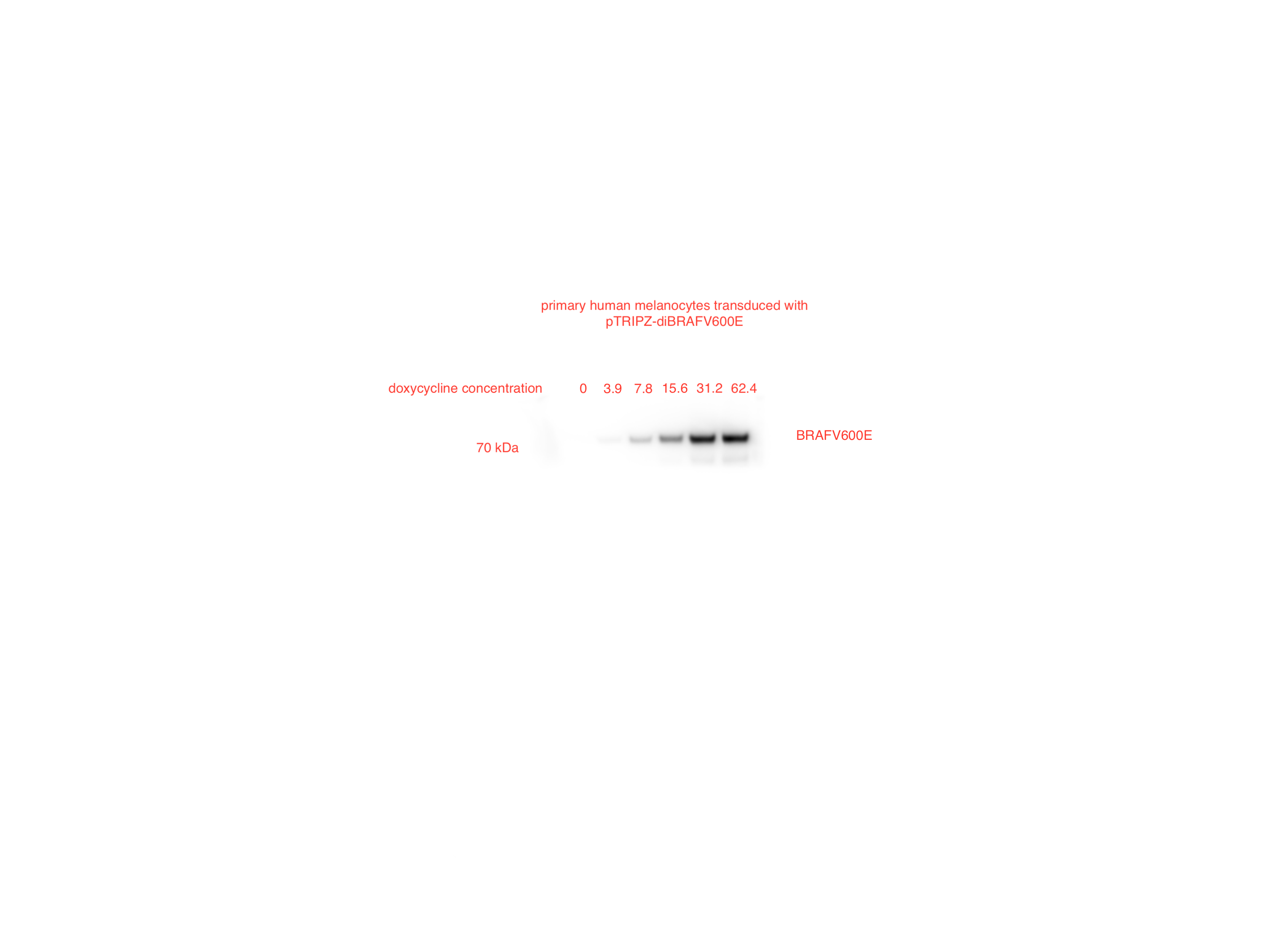

Supplement: Figure 3—source data 1. [file elife-70385-fig3-data1.zip › dibve_wb/figure_3A_source_data_BRAFv600e_annotated.tiff]

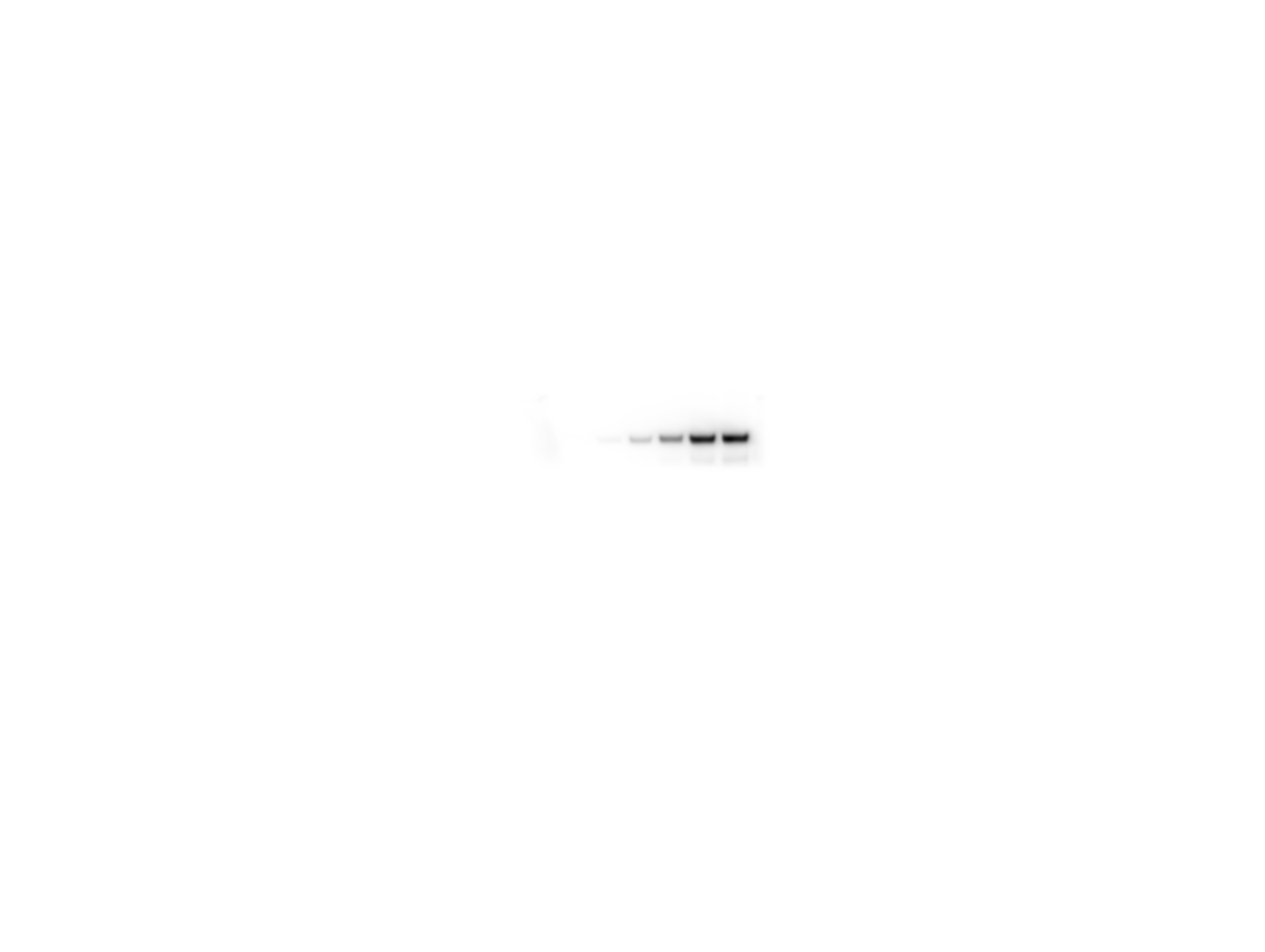

Supplement: Figure 3—source data 1. [file elife-70385-fig3-data1.zip › dibve_wb/figure_3A_source_data_BRAFv600e_pub.tif]

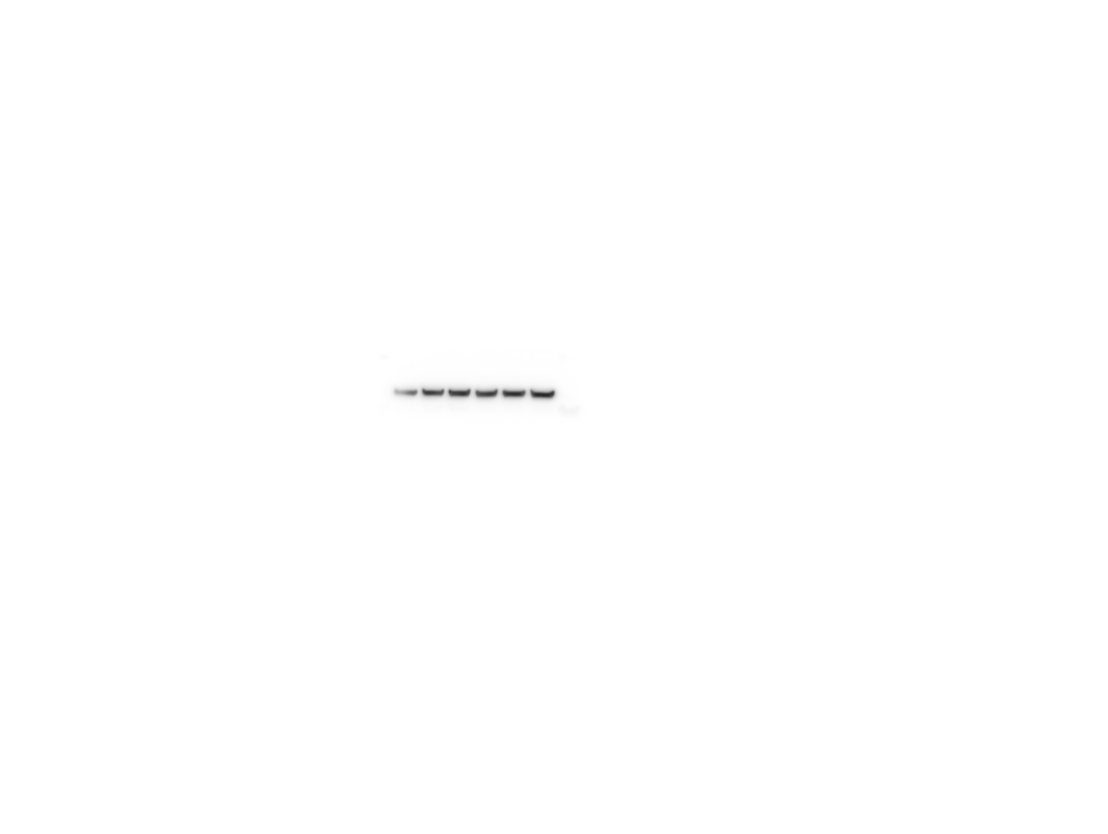

Supplement: Figure 3—source data 1. [file elife-70385-fig3-data1.zip › dibve_wb/figure_3A_source_data_hsp90.tif]

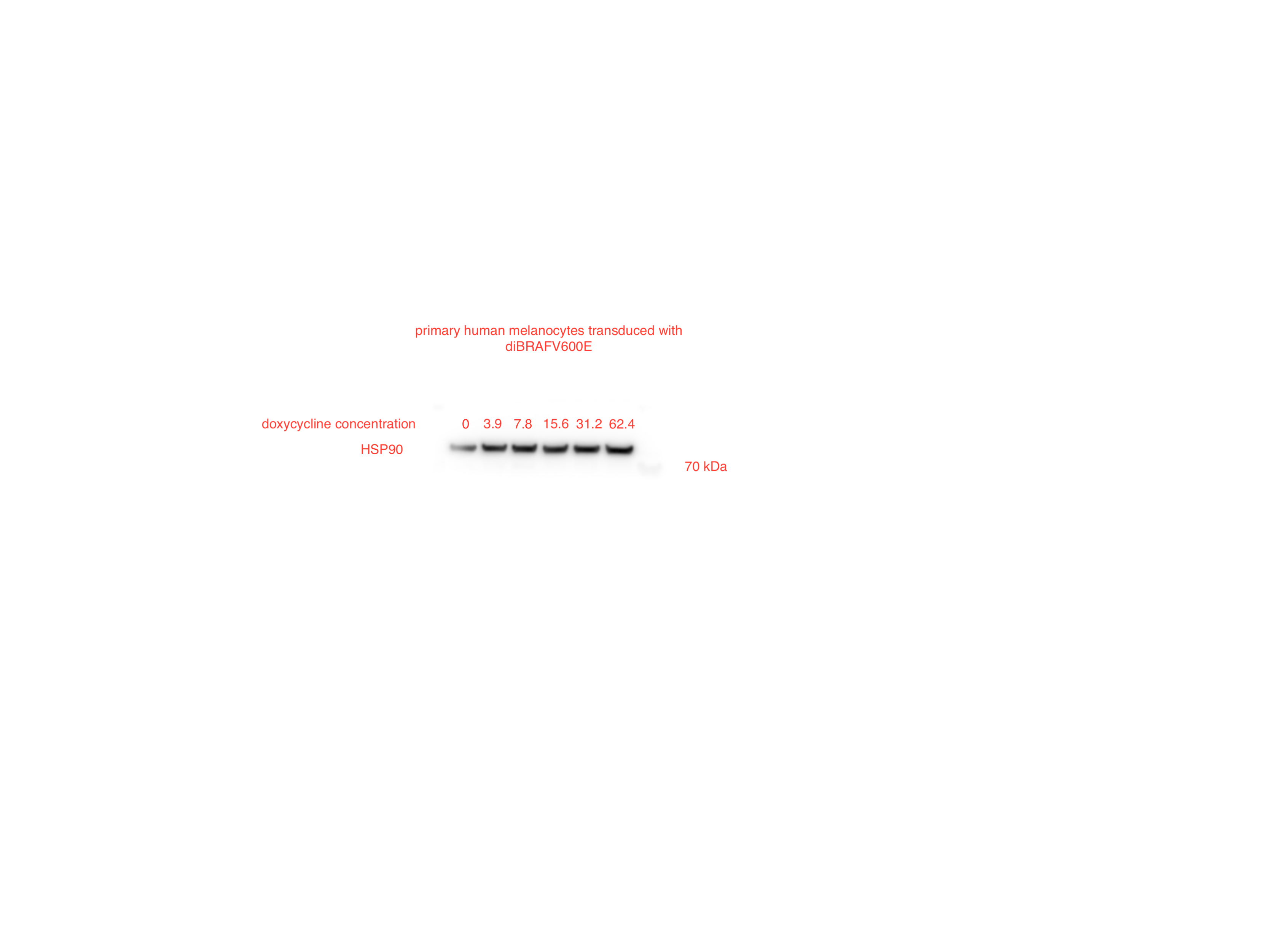

Supplement: Figure 3—source data 1. [file elife-70385-fig3-data1.zip › dibve_wb/figure_3A_source_data_hsp90_annotated.tif]

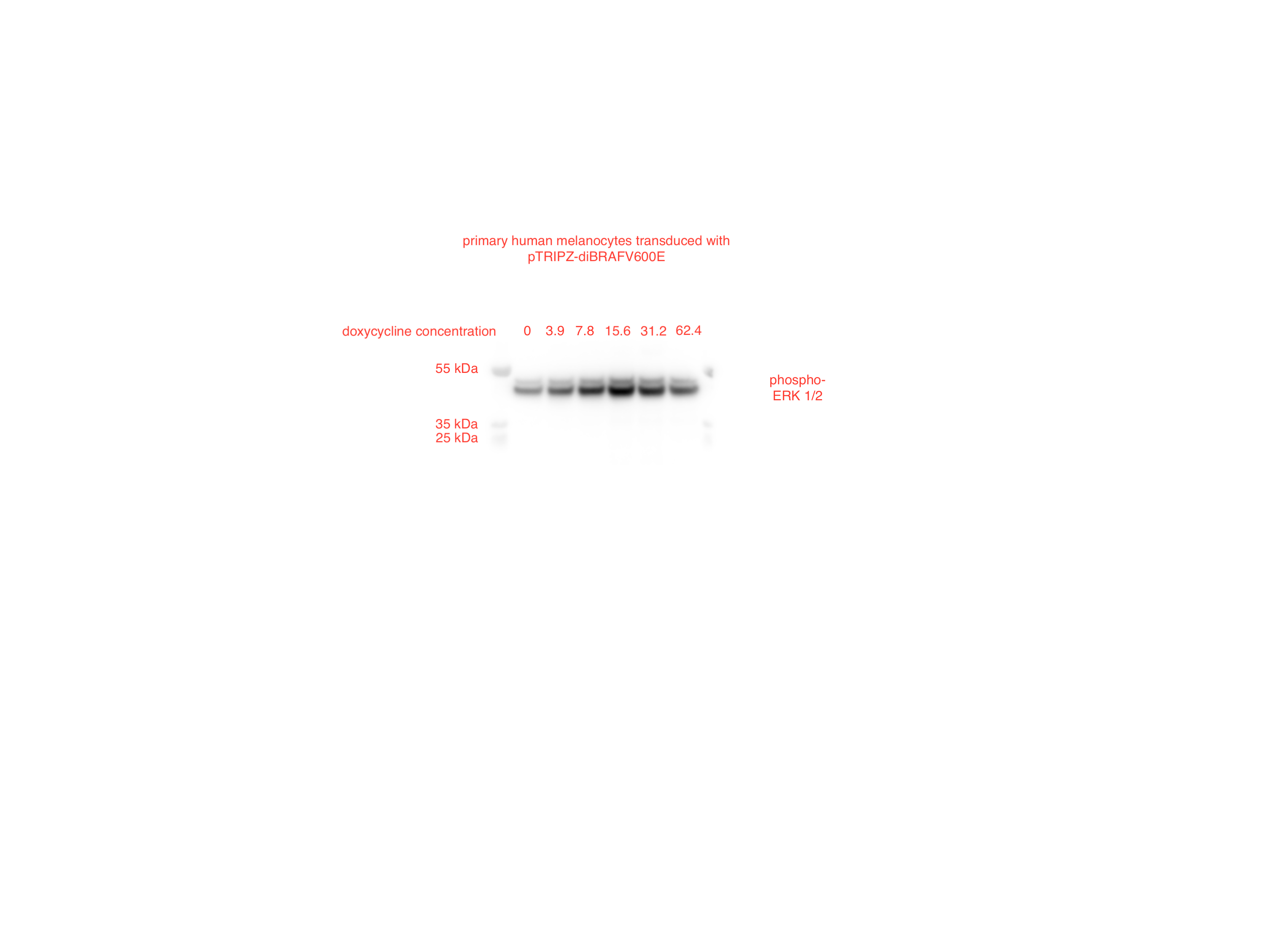

Supplement: Figure 3—source data 1. [file elife-70385-fig3-data1.zip › dibve_wb/figure_3A_source_data_phospho_erk_annotated.tiff]

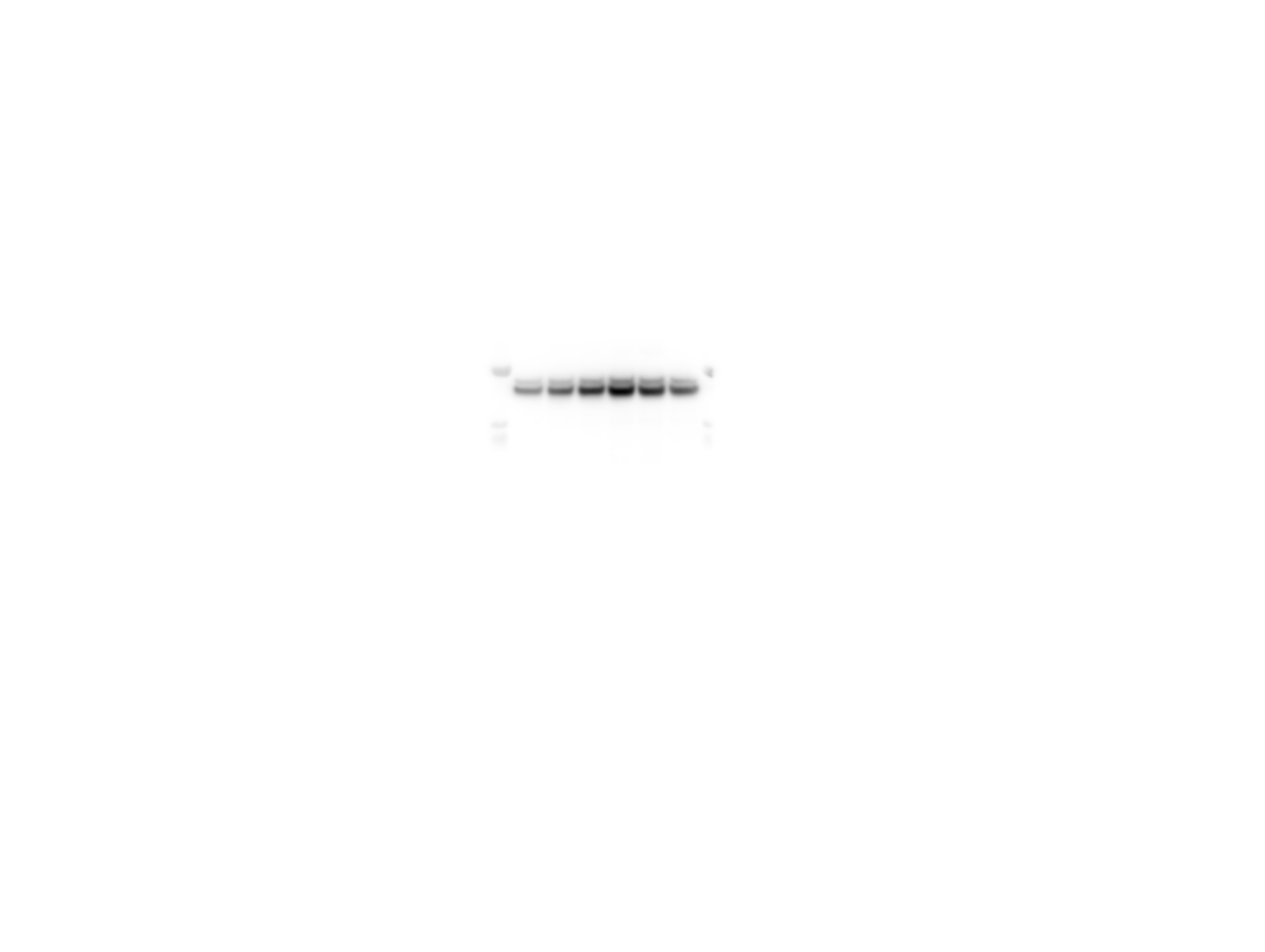

Supplement: Figure 3—source data 1. [file elife-70385-fig3-data1.zip › dibve_wb/figure_3A_source_data_phospho_erk_pub.tif]
